# Is there a significant relationship between the empowering leadership behaviors of school principals and the psychological resilience of teachers? Understanding the moderating effects of gender and length of time spent with the school principal

**DOI:** 10.1186/s40359-024-02330-3

**Published:** 2025-01-08

**Authors:** Mehmet Sabir Çevik, Emine Doğan

**Affiliations:** 1 Siirt University, Siirt, Türkiye; 2 Kahramanmaraş Sütçü İmam University, Kahramanmaraş, Türkiye

**Keywords:** Empowering leadership, Psychological resilience, School principals, Moderating effect

## Abstract

The psychological resilience of teachers has gained significant attention due to its positive effects on schools and students. This cross-sectional quantitative study investigates the moderating roles of gender and the length of time spent with the school principal in the relationship between principals' empowering leadership behaviors and teachers' psychological resilience. Data were collected from 362 teachers working in public primary, secondary, and high schools in Siirt, Türkiye. Descriptive statistics, correlation analysis, and moderation analysis using the bootstrapping method were applied to analyze the data. The findings reveal that the empowering leadership behaviors of school principals positively correlate with teachers' psychological resilience, with variations based on gender and time spent with the principal. Specifically, the relationship is stronger for female teachers compared to male teachers and for those who have spent more time with the school principal. These results offer theoretical insights and practical recommendations for enhancing teacher resilience through leadership practices, highlighting the importance of understanding contextual and demographic factors.

**Supplementary Information:**

The online version contains supplementary material available at 10.1186/s40359-024-02330-3.

## Introduction

In Türkiye and many other countries, many problems such as high levels of job stress, lack of professional autonomy, disadvantaged schools, and student or parent bullying negatively affect teachers psychologically [1–5]. Additionally, incidents of violence against teachers, which have been frequently discussed in the written and visual media recently [6, 7] and the feeling that the teaching profession is not valued by society [8] reduce the resilience of teachers, leading to negative consequences at individual, organizational, and social levels [9]. Psychologically exhausted teachers may leave the profession after practicing it for a few years. In some countries (e.g., the United Kingdom), it is reported that 30–50% of teachers leave the profession in their first years of teaching [10, 11]. A more important problem is that there are teachers who continue their profession without psychological resilience (PR). This situation can negatively affect more than one class and development of students [12]. Therefore, it is very important to empower teachers [13, 14], who are frequently emphasized to have a key role in school and student success [15–17]. It is also known that empowerment efforts of school principals also increase teacher performance [18]. All these implications point to the need for empirical research on determining school principal leadership styles strengthening psychological resilience of teachers.

As a result of the adoption of teacher empowerment as a school management strategy, there has been an increasing interest from academicians and practitioners in how school leaders can empower teachers more effectively [19–24]. Bolden et al. [25] state that the right leadership style can meet the great need to create psychological resilience in teachers. As resilience levels of teachers increase, they are expected to cope better with undesirable behaviors of their students, be less affected by negative conditions in their work environment, and be more successful in coping with difficulties [26]. Peters and Pearce [27] conducted a qualitative study that aimed to uncover the conditions fostering resilience of teachers at the beginning of their careers and discovered that school leaders played a central role in providing formal and informal support. Empowering leadership (EL) is highly relevant to such situations, because it focuses on encouraging self-management and removing the constraints of powerlessness [28].

If the school principal, as an empowering leader, gives responsibility and authority to the teachers who are in closest contact with the students and supports their work, this will be regarded as a positive behavior by the teachers and will contribute to obtain more effective results [29]. Empowering leadership enables teachers to be more intrinsically motivated and professionally committed to their work roles [15, 30–33]. Therefore, there are empirical research findings that empowering leadership psychologically strengthened its followers [34]. In other words, when school principals support teachers, grant authority by sharing power, and exhibit empowering leadership behaviors encouraging teachers to be aware of their responsibilities, these behaviors can contribute to the psychological strengthening of teachers and thus increase their psychological resilience.

Previous literature has expanded the literature demonstrating the importance of leadership in terms of teachers’ emotional states by proving the positive relationship between teacher resilience and school leadership [35, 36]. In the literature, there are also studies showing that psychological resilience plays a mediating role between school leadership and teacher empowerment [22]. Additionally, existing literature has also proven the positive effect of empowering leadership on emotional states of teachers such as psychological resilience, psychological capital, and psychological well-being [37–40]. Moreover, some studies report that empowering leadership contributes to school development through emotional states of teachers [41]. For example [42] found that empowering leadership increased teachers' commitment through their subjective well-being. Dash and Vohra [43] showed that empowering leadership plays a mediating role between teachers' alienation and emotional commitment. The literature review revealed that there is a lack of research and uncertainty regarding the moderating effect between empowering leadership and teacher resilience. Therefore, more studies are needed to understand the conditions under which empowering principal behaviors strengthen or weaken teacher resilience. In order to address the gaps and eliminate ambiguity in the relevant literature, the current study aimed to examine the relationship between the empowering leadership of school principal and psychological resilience of teachers by focusing on the moderating role of teacher gender and the length of time they work with school principals.

In educational leadership studies, the gender variable is generally outside the scope of research [44]. This is concerning because gender is considered as one of the key structural variables affecting leadership perceptions [45]. On the other hand, there is uncertainty in the literature regarding the relationship between length of time spent with the school principal and leadership behaviors. For example, while one study reported that length of time spent with the school principal eroded relationships [46], another study reported that it increased interaction [47]. The literature review revealed a lack of research specifically addressing the moderating effects between empowering leadership and psychological resilience of teachers. Based on this, this article aims to examine the effects of empowering leadership behaviors of school principals on psychological resilience of teachers and offer a new perspective by focusing on the moderating effects of gender and length of time spent with the school principal. The study findings may provide important implications for the leadership style that school administrators should adopt and may guide theoretical frameworks in subsequent research. Additionally, understanding the leadership style and some demographic characteristics influencing teacher psychological resilience may be vital in determining effective intervention and prevention strategies.

## Study context: education system and school leadership in Türkiye

In Türkiye, 48.7% of teachers stated that they were exposed to violence, and 94.1% stated that they were psychologically worn out because of their profession [48]. Similarly, some studies conducted in Türkiye also show that violence against teachers is common. Özkılıç [49] found that 40.9% of the participants in his study were subjected to violence, [50] found that 50% of them were subjected to violence. Albeit limitedly, the policies implemented in Türkiye in recent years aim to strengthen the teaching profession. In this regard, the Teaching Profession Law, which came into force in 2022, and the Teacher Strategy Paper, which came to the agenda in 2018, attract attention [51, 52]. With these and similar regulations, it is aimed to increase the social influence and prestige of the teaching profession. However, the implementation process in Türkiye is painful for teachers.

Türkiye has a highly centralized and hierarchical education system in which MoNE (Ministry of National Education) is responsible for the planning and control of all tools and processes [53]. As it is known, differences in status, rank, authority, social position, and power in hierarchy can often turn into physical, social, and psychological distances, which can affect the degree of social closeness that develops [54, 55]. Buluç [56] states that excessive centralization and bureaucratic structure are obstacles to leadership for school principals. This situation make school principals adopt a bureaucratic management approach and adhere to strict norms. For example, empowering leadership requires some authority to be delegated to teachers, but current legislation places restrictions on school principals’ power to delegate authority. However, in Türkiye, a policy document has recently been published that aims to build school leadership capacity to strengthen school improvement efforts [57]. Researchers state that these policy initiatives are a strategic support for teachers [58, 59]. Although policy makers focus on the changing roles of school principals and the effectiveness of teachers in teaching processes, empirical evidence on the impact of principal leadership practices on teacher resilience in Türkiye is too limited to inform and guide this policy trend. Our findings on the effectiveness of strengthening school leadership in the Turkish case, in line with recent reform movements, are important. Because there are problems regarding whether Western leadership models are applicable in Asian cultures [60]. In this context, it can be understood that it is very important that school principals in Türkiye support autonomy of teachers by giving them responsibility and authority with an empowering understanding.

The study data were collected from the Siirt province of Türkiye. Siirt is one of the provinces in the “Third Service Area” in the southeast of Türkiye, where teacher circulation (labor turnover) is high. Teacher circulation is an important educational problem throughout Türkiye, especially in the eastern and southeastern regions, which are considered disadvantaged regions. In order to solve this problem, some measures such as compulsory service are taken. MoNE follows various policies in teacher appointments in order to control teacher circulation and balance the mobility in compulsory service regions with the application of compulsory service regions and compulsory service years [61]. When we look at the literature, the difficulties faced by teachers at the beginning of their careers in many countries are frequently mentioned, and reports of teacher stress and burnout are confirmed [62, 63]. A significant portion of the teachers working in the province of Siirt, where our study was conducted, were newly appointed to the teaching profession and were working in disadvantaged schools. Additionally, the average education level is quite low in Siirt province, which is located in the Southeastern Anatolia Region, where the education disparities between regions are the highest [64]. Teachers working in Siirt province were specifically selected for the study because it is one of the provinces where empowering leadership behaviors of school principal are needed the most for the psychological resilience of teachers. Moreover in the Turkish society with a patriarchal structure [65] and the Turkish education system where school administrators can work at a school for a maximum of eight years [66], the moderating effect of gender and the length of time spent with the school principal on the relationship between empowering leadership and psychological resilience of teachers can provide significant insights to practitioners and policy makers.

## Development of theoretical framework and hypotheses

Theoretically, the contribution of empowering leadership to increasing subordinates' resilience can be explained by Social Exchange Theory (SET). Blau [67] social exchange theory states that the relationships between the organization's employees and the organization are a kind of change and the expectation is mutual. Social exchange theory can be defined as an individual feeling obliged to reciprocate in the same way to someone who treats him well [68]. Social exchange theory states that a high-quality leader-member exchange relationship will increase effectiveness of employees [69]. This process is explained by the basic principle of social exchange theory called the “norm of reciprocity.” The norm of reciprocity is the expectation that an individual who experiences positive behavior from another will respond positively to this behavior [70]. In the context of social exchange theory, it is based on the norms of social exchange and reciprocity that empowering leaders empower employees and offer expanded authority and responsibility to increase organizational success. An empowering leader can influence the resilience of employees by carrying out activities increasing the social change of the organization. In other words, if leaders are perceived as supportive, increasing intrinsic motivation, distributing their power and authority to employees, making employees feel autonomous, this will enable employees to be more resilient to challenges. On the contrary, when employees are not supported, not motivated, not provided with an area where they can develop their skills, not made to feel autonomous, and not provided with open communication and information sharing, they are likely to exhibit negative behaviors.

There are strong conceptual connections between the actions of an empowering leader and psychological empowerment [71]. Accordingly, school principals’ provision of autonomy and authority will lead to a social exchange, create an environment of trust, and increase the level of resilience of teachers. Additionally, Social Exchange Theory emphasizes that individuals are more giving when they receive something they expect to receive in return. School principals’ emotional support of teachers and their empowerment with positive incentives will increase teacher performance and positively affect school and student success. In this study, in addition to exploring the relationship between empowering leadership and teacher resilience utilizing the benefit-reciprocity assumption of SET, we also explain how this relationship may emerge by incorporating the positive organizational behavior (POB) perspective into SET. Researchers studying POB include adopting a positive approach to the study of organizational behavior [72–74]. Similarly, organizations positively affect employee performance by focusing on positive personal development [75, 76]. In this context, in our study, we based the POB perspective on positive personal development within the framework of the SET assumption.

## Psychological resilience and empowering leadership

The word resilience comes from Latin and means “to bounce back” and recover from bad times as soon as possible [77]. Resilience is, on the one hand, overcoming difficulties and, on the other hand, not losing the ability to develop social, academic, and professional competence despite stressful situations [78]. Psychological resilience is defined as “the ability to make progress even in high-risk situations, to overcome trauma quickly, to maintain competence even when exposed to stress, and to cope with dynamic changes” [79, 80]. Studies show that employees who can overcome difficulties and failures and move on have higher job satisfaction, organizational commitment, and job performance, and are more effective in their jobs [81, 82]. Therefore, psychological resilience is identified as a non-cognitive ability and a desirable trait for teachers [83]. Resilient teachers are described as those having a strong sense of self-confidence, hope, optimism about the future, and who learn from past failures and overcome negative experiences [78]. As can be understood from these explanations, psychological resilience, which affects the cognitive and affective processes of the individual and has the power to shape organizational behaviors positively, can be considered as a variable that can affect the effectiveness of education for teachers. In this study, the psychological resilience conceptual framework of [84] was adopted.

Empowering leadership was first introduced into the literature by [85] and is defined as “super leadership” as leaders who direct their followers to self-management [86]. The concept of empowerment, which forms the theoretical basis of empowering leadership, refers to “practices and situations in which individuals feel motivated, can take risks, are motivated to participate in decisions, believe that they can control events, and their confidence in their knowledge and expertise increases,” [87, 88]. Empowering leadership constitutes the behavioral dimension of empowerment [89]. It is the process of distributing power, autonomy, and responsibility to employees in order to increase their intrinsic motivation and organizational success [34, 90]. In this study, the conceptual framework created by [89] was adopted. Konczak [89] categorized empowering leadership behaviors as empowerment, responsibility, self-determination, knowledge sharing, skill development, and coaching for innovative performance. The framework created by [89] was shaped as *empowerment, responsibility, and support* in the study adapted to Turkish culture in educational institutions by [91]. *Empowerment* includes leaders providing opportunities for expert/competent employees to make their own decisions in order to improve the quality of work processes; *responsibility* includes leaders giving employees the necessary responsibility to develop their own actions in their field of activity and create new actions; and the *support* dimension includes behaviors such as leaders providing employees with control through information sharing, mentoring, and encouraging them for professional development.

School principals who have empowering leadership characteristics such as giving teachers responsibility, authority, and support convey trust in teachers, empower them, and make them more resilient [23]. In studies conducted in educational organizations, school principals, as empowering leaders, significantly affect the well-being of teachers [41]. Moreover, in educational organizations, empowering leadership is associated with positive organizational behaviors such as self-efficacy [92], organizational commitment [15, 18, 20], organizational citizenship [31], innovative behaviors, and innovative climate perceptions [17, 33, 93] of teachers. Similarly, empowering leadership affects teachers psychologically positively. Empowering leadership is positively associated with psychological empowerment [37], psychological contract perceptions [38], and psychological well-being [40] of teachers; while it is negatively associated with their professional burnout [29], intention to leave work [94], and alienation [43]. Based on the results of existing studies in the literature, Hypothesis 1 regarding the significant and positive effect of empowering leadership behaviors of school principals on psychological resilience of teachers is presented below.

H1: There is a positive significant relationship between empowering leadership behaviors of school principals and psychological resilience of teachers.

### Gender and length of time spent with the school principal as moderating variables

The gender variable can enable the discovery of important findings in empirical research. According to [95], women are more prone to emotional contagion than men, both for positive and negative emotions. Therefore, it can be said that women are more affected by the emotional atmosphere around them than men. The fact that studies show that female teachers tend to score higher on issues such as fatigue and burnout, while male teachers are less likely to be negatively affected psychologically by similar events confirms this discourse [96, 97]. Buttner [98] found that in leader–follower relationships, women are more concerned with task-related support (such as encouraging task participation and inquiry, resource allocation support, and providing help with problem solving), while men are more concerned with outcomes. As a result, empowering leadership behaviors of school principals may be more effective on psychological resilience of female teachers. Examining employee resilience, [99] recommended conducting further research that could include investigating the effects of gender differences on resilience and examining the moderating role of gender in the influence of leadership on follower resilience. In this context, Hypothesis 2 regarding the moderating effect of gender is presented below.

H2: Gender has a moderating role in the relationship between empowering leadership behaviors of school principals and psychological resilience of teachers. In other words, the effects of empowering leadership behaviors of school principals on psychological resilience of teachers differ between male and female teachers.

Because it takes time to form leader-employee relationships in organizations, academicians argued that time spent together can be an important moderating factor [100]. According to [101] filter theory, as individuals get to know each other more closely over time, superficial ties between them turn into deeper ties. This argument is based on the premise that as interpersonal communication deepens, individuals better understand each other's values, beliefs, and experiences [100]. However, this theory may not be valid in all cases. For example, a study in Spain found that the longer the principals remained in office, the less motivated teachers were [46]. Although the findings of the study conducted by [102] in the Turkish sample showed that the length of time spent with the school principal did not create a significant difference in empowering leadership perceptions of the teachers, the moderating effect of the length of time spent with the school principal between empowering leadership and psychological resilience of teachers is still not fully known. The relevant literature suggests that the length of time teachers spend with the school principal may influence the positive relationship between empowering leadership and psychological resilience of teachers. Hypothesis 3 based on this claim is presented below.

H3: The length of time spent with the school principal has a moderating role in the relationship between empowering leadership behaviors of school principals and psychological resilience of teachers. In other words, the effects of empowering leadership behaviors of school principals on psychological resilience of teachers differ between teachers with low and high length of time spent with the school principal.

In conclusion, this study, based on a theoretical framework supported by empirical data and developed with a quantitative understanding, suggests that there is a positive relationship between empowering leadership style of school principals and psychological resilience of teachers, and that gender and length of time spent with the school principal also have moderating effects on this relationship. Examining the relationship between the mentioned variables together with their moderating effects may contribute empirically to a better understanding of the teacher psychological resilience issue in the literature. The empowering leadership approach that includes teacher empowerment not only increases psychological resilience of teachers, but also contributes to the creation of a more effective and successful educational environment. It can also serve as a reference point for policy makers who want to have a positive impact on the development of education processes.

## Method

This is a quantitative study conducted with a cross-sectional and moderator analysis design. Cross-sectional studies involve collecting data in a single time period in order to make inferences about the studied universe [103]. Moderator analysis reveals how the effect of a predictor variable on another predicted variable is moderated [104]. This study is a cross-sectional one as data were collected in a single time period. Moreover, moderator analysis was also used in the study to determine which variables affect the effect of the predictor variable on the predicted variable. Accordingly, in the study, the empowering leadership behaviors of school principals were defined as the predictor variable, the psychological resilience of teachers as the predicted variable, and gender and length of time spent with the school principal as the moderating variable. In the study, the variable related to the school, namely the level of education, and the variable related to personal characteristics of the teachers, namely educational status and marital status, were analyzed as control variables.

### Sample and data collection process

In this study, the target audience was defined as teachers working at primary, secondary, and high school levels in Siirt city center in the spring semester of the 2023–2024 academic year. While the universe of the study covers these 2322 teachers, the sample consists of 330 teachers determined by simple random sampling method at 95% confidence level. The simple random sampling method aimed to minimize the risk of bias by ensuring that each individual in the universe had an equal probability of being selected for sampling. The simple random sampling method was also preferred in order to increase the generalizability of the study findings to the universe. It was also analyzed whether the demographic characteristics of the sample (gender, marital status, level of education, teaching stage, and length of time spent with the school principal) were compatible with the general characteristics of the universe and it was found that they were compatible. In other words, the fact that the participants of the study were teachers working at different levels of education and with diverse demographic characteristics ensured that the study results could be generalized and represented. Additionally, in this study, some selection criteria were applied to determine the teachers to be included in the sample. Teachers who would participate in the study had to be working in primary, secondary, and high schools in Siirt city center, be actively working in the spring semester of the 2023–2024 academic year and agree to participate in the study voluntarily. These selection criteria were determined to create a sample appropriate for the purpose of the study and to increase the reliability of the data obtained.

In this study, the data collection process was carried out in accordance with the ethical standards of the study. Firstly, the universe consisting of teachers working in primary, secondary, and high schools in Siirt city center was defined. Direct contact was established with school principals to reach the participants. With the permission of the school administrations, the scale forms were delivered to the teachers online via WhatsApp and e-mail. The participants were informed that the necessary ethical permissions for the study had been obtained, that the study did not carry any risks, that the study would be conducted on a voluntary basis, that the collected data would be examined anonymously and would not be used for purposes other than scientific. Moreover, contact information of the researchers was shared in order to answer questions from teachers and explain unclear parts throughout the data collection process. The online scale form consisted of 23 questions, in addition to the questions about the school (level of education) and the demographic characteristics of the teachers (gender, marital status, educational status, length of time spent with the school principal). In the scales, in order to reduce method bias and social desirability, questions were first asked about the dependent variable (psychological resilience) and then about the independent variable (empowering leadership), and anonymity was maintained throughout the study period [105]. However, the response rate was 94%, and 376 teachers out of 400 filled out the scale form. Of 376 data of the study, 14 data that gave the same answer to all items, did not show any diversity, and were seen as extreme values ​​were not included in the analysis, and as a result, analyses were carried out with 362 data. In this context, 192 (53%) of the teachers participating in the study were male, 170 (70%) were female; 109 (30.1%) were, and 253 (69.9%) were married. Of the teachers, 324 (89.5%) had a bachelor's degree, 38 (10.5%) had a postgraduate degree; 104 (28.7%) worked in primary school, 112 (30.9%) in secondary school, and 146 (40.3%) in high school. It was also determined that the length of time the teachers spent with the school principal varied between 1 and 7 years, and the average length of time spent with the school principal was 2.36 (SD = 1.52).

### Data collection tools and variables

#### Independent (Predictor) Variable: Empowering Leadership Scale (ELS)

In the study, the “Empowering Leadership Scale (ELS)” developed by [89] was used to measure the empowering leadership behaviors of school principals. The ELS is a five-point Likert-type scale ranging from 1 (never) to 5 (always) and consists of six sub-dimensions and 17 items. In this study, the three-dimensional structure of the scale adapted to Turkish by [91] was used. The sub-dimensions of “self-determination, knowledge sharing, skill development, and coaching for innovative performance”, which emerged as different dimensions in the study by [89], were combined into a single dimension called “support” in Turkish adaptation study by [91]. In this context, the Turkish adaptation of the scale consists of the sub-dimensions of “empowerment (Items 1, 2, and 3. Sample item: *My school principal gives me authority equal to my responsibility in the matters s/he assigns me*), responsibility (Items 4, 5, and 6. Sample item: *My school principal holds me responsible for the work I am assigned to do*), and support (Items 7, 8, 9, 10, 11, 12, 13, 14, 15, 16, and 17. Sample item: *My school principal frequently provides me with opportunities to develop new skills*)”. As a result of the Turkish adaptation of the ELS, it was reported that the Cronbach alpha coefficients for the sub-dimensions of empowerment, responsibility, and support were 0.76, 0.82, and 0.80, respectively, and the results of the confirmatory factor analysis (CFA) conducted to determine the validity of the scale structure were within the appropriate value ranges (*x*^2^/sd = 2.54; GFI = 0.92; NNFI = 0.98; CFI = 0.95; RMSEA = 0.054; SRMR = 0.032) [67]. For the current study, reliability and validity analyses of the ELS were re-conducted. In this context, it was determined that the Cronbach alpha and composite reliability (CR) coefficients for the sub-dimensions of empowerment, responsibility, support, and the entire scale were above 0.70, and the average variance value explained (AVE) was above 0.50 (see Table 1). On the other hand, we found that the SRW (Standardized Regression Weights) values ​​of all items related to the scale were above 0.50. This result means that the construct validity of the scale is strong [106, 107]. Moreover, it was determined that the fit indices regarding the first-order CFA analysis results showing the fit of ELS with the study data were within statistically appropriate ranges (*x*^2^/sd = 3.15; RMSEA = 0.077, SRMR = 0.039, CFI = 0.96, TLI = 0.95).

**Table 1:** Correlation between descriptive statistics and variables (*n* = 362)

Variables	M	SD	AVE	α	CR	EL	EMP	RES	SUP	PR
EL	3.38	0.82	0.72	0.95	0.97	-				
EMP	2.66	1.00	0.72	0.88	0.88	.668^**^	-			
RES	3.61	1.00	0.86	0.95	0.95	.688^**^	.433^**^	-		
SUP	3.51	0.95	0.69	0.96	0.96	.957^**^	.486^**^	.514^**^	-	
PR	3.53	0.92	0.75	0.95	0.95	.687^**^	.453^**^	.628^**^	.615^**^	-

*EL* Empowering Leadership, *EMP* Empowerment, *RES* Responsibility, *SUP* Support, *PR* Psychological Resilience, *M* Mean, *SD* Standard Deviation, *AVE* Average Variance Explained, *α* Cronbach Reliability Coefficient, *CR* Composite Reliability Coefficient

^**^p < .01

#### Dependent (Predicted) Variable: Brief Psychological Resilience Scale (B-PRS)

In the study, the Brief Psychological Resilience Scale (B-PRS), developed by [84], was used to measure the psychological resilience levels of teachers. The Turkish adaptation of B-PRS was conducted by [108]. The scale is a five-point Likert-type (1: Not at all appropriate, 5: Completely appropriate), six-item (Sample item: *I can quickly pull myself together after difficult times.*) measurement tool consisting of a single sub-dimension. The 2nd, 4th, and 6th items of the scale are reverse items. The lowest score that can be obtained from B-PRS is 6, and the highest score is 30. A low score obtained from the scale indicates low psychological resilience while a high score indicates high psychological resilience. The Cronbach alpha coefficient of the scale is 0.83. It was stated that the CFA results (*x*^2^/sd = 1.83, NFI = 0.99, NNFI = 0.99, CFI = 0.99, IF = 0.99, RFI = 0.97, GFI = 0.99, AGFI = 0.96, RMSEA = 0.05, SRMR = 0.03) were within the appropriate ranges [89]. For this study, it was determined that the Cronbach alpha and composite reliability (CR) coefficients of the scale were above 0.70, and the average variance value explained (AVE) was above 0.50 (see Table 1). Additionally, we found that the SRW values ​​of all items on the scale were above 0.50. This result means that the construct validity of the scale is strong [106, 107]. Moreover, within the scope of the current study, it was seen that the fit indices of B-PRS were also suitable according to the first-order CFA analysis (*x*^2^/sd = 4.48; RMSEA = 0.098, SRMR = 0.016, CFI = 0.99, TLI = 0.98).

#### Moderating and control variables

Based on previous empirical studies [109–113], in our study, gender of teachers and length of time spent with the school principal were analyzed as moderating, and their level of education, marital and educational status were analyzed as control variables.

## Data analysis

The study data were reported using Mplus 8.3 [114] and SPSS Process Macro 3.5.3 [115] programs. The descriptive statistics of the study, the relationships between the variables, and the CFA results (H1) were tested with M*plus* 8.3 while the moderator analyses regarding the H2 and H3 hypotheses were tested with Model 1 of the Process Macro models. In the study, 95% confidence intervals were calculated for the moderator effect according to the bootstrapping method with 5000 resample. The significance of the 95% bootstrapping confidence interval was evaluated according to whether it did not contain the zero value [116]. When the moderator effect was significant, simple slope analysis graphs were drawn to understand the results of the moderator effect more clearly [117]. In order to determine the compatibility of the scales used in the study with the data, chi-square/degree of freedom (x^2^/df), RMSEA, SRMR, CFI, and TLI values ​​were taken into account. In these compatibility values, x^2^/df being less than 5, SRMR being less than 0.05, RMSEA being less than 0.10, and TLI and CFI being greater than 0.90 means that the scale is compatible with the data [118–120].

In the study, missing and outlier (extreme) values ​​of the data were examined before performing the analyses. Since the data were collected online, no missing values ​​were found. Z scores were calculated to determine the outliers (extreme) of the data, and 14 data that were outside the range of -3 and + 3 and gave the same answer to all items or did not show any diversity were excluded from the analysis. The study data were also checked to see whether they met the assumptions for the necessary analyses. In this context, it was decided whether the data showed a normal distribution by examining the skewness and kurtosis values. As a result of the examination, it was determined that the skewness value of the empowering leadership behaviors score was -0.182, and the kurtosis value was -0.418; the skewness value of the psychological resilience score was -0.465, and the kurtosis value was -0.164. Skewness and kurtosis values ​​between -1.5 and + 1.5 indicate that the data have a normal distribution [121]. Moreover, it was detected that the points in the Q-Q charts were close to the 45-degree line, and the data showed a distribution in accordance with the multivariate normality assumptions based on the multivariate scatter diagrams.

Tolerance, VIF (Variance Increase Factors Method), CI (Conditional Index Numbers Method), and Durbin-Watson values ​​were examined to determine whether there was a multicollinearity problem among the variables of the study. As a result of the analyses carried out for this purpose, it was seen that the Tolerance values ​​of the study data varied between 0.984 and 0.997; VIF values ​​varied between 1.003 and 1.016; CI values ​​varied between 3.828 and 12.664, and the Durbin-Watson value was 1.911. In the current study, the fact that the Tolerance value was greater than 0.20, VIF value was less than 10, CI value was less than 30, and Durbin-Watson value was between 1.5 and 2.5 means that there is no multicollinearity problem [122–124]. Finally, the fact that the data were cross-sectional and collected only from teachers may cause common method bias. Therefore, Harman's single-factor test was applied to detect common method bias in the study [125, 126]. Varimax rotation factor analysis was performed with a total of 23 scale items regarding the variables of the study, and it was concluded that the analysis results were not collected under a single factor. Moreover, as a result of varimax analysis, we found that the four factors with eigen values ​​over 1.00 explained 78.55% of the total variance, and the variance explained by the first factor was less than 50% (33.60%). All these results can be considered as evidence that there is no common method bias in the study.

## Findings

In this section, before presenting the findings related to each hypothesis, descriptive statistics and correlation analysis findings are given.

### Findings regarding descriptive statistics and correlation analysis

In the study, firstly, the descriptive statistics, reliability coefficients, and Pearson correlation analysis findings are presented (see Table 1). It was determined that the Cronbach alpha coefficients for the scales and their sub-dimensions were above 0.70. In terms of convergent validity, the composite reliability (CR) coefficient was found above 0.70. Additionally, the AVE value being above 0.50 and the CR coefficients being higher than the AVE values ​​is evidence that convergent validity was achieved [127]. Consequently, the fact that the reliability coefficients in the study were above 0.70, CR was greater than AVE, and AVE was above 0.50 means that the scales of the current study are reliable and valid [128–130].

In terms of the mean values ​​of the study, it was determined that empowering leadership (M = 3.38, SD = 0.82) interventions were at a medium level close to high level; empowerment (M = 2.66, SD = 1.00) interventions were at a medium level. It was determined that responsibility (M = 3.61, SD = 1.00), support (M = 3.51, SD = 0.95), and psychological resilience (M = 3.53, SD = 0.92) had high mean values. On the other hand, teachers' perceptions of psychological resilience in terms of SD (Standard Deviation) showed a greater deviation than the empowering leadership variable. Additionally, a moderately positive significant relationship was found between empowering leadership and psychological resilience (*r* = 0.687; *p* < 0.01). This finding supports the H1 hypothesis of the study. Furthermore, the relationships between the sub-dimensions of empowering leadership and psychological resilience were significantly moderate and positive (*p* < 0.01).

### Findings regarding testing hypotheses

To test the moderating role of gender in the effect of empowering leadership behaviors of school principals on psychological resilience of teachers, regression analysis based on the bootstrap method was conducted. 5000 resampling options were used with the bootstrap method. Accordingly, the regression analysis findings showing whether the effect of empowering leadership on psychological resilience varies according to gender are given in Table 2.

**Table 2:** Regression analysis findings regarding the moderating effect of gender (*n* = 362)

Variables	*b*^*a*^	S.E	t	p	95% Confidence Interval	
					Minimum	Maximum
Constant	3.839	0.239	16.048	0.000^*^	3.369	4.309
EL	0.479	0.133	3.605	0.000^*^	0.218	0.740
Gender	-0.046	0.071	-0.640	0.522	-0.185	0.094
EL x Gender	0.196	0.086	2.290	0.023^**^	0.028	0.365
Level of Education	0.009	0.044	0.202	0.840	-0.077	0.095
Educational Status	-0.148	0.116	-1.278	0.202	-0.375	0.080
Marital Status	-0.058	0.079	-0.731	0.465	-0.213	0.097

*S.E.* Standard Error, *EL* Empowering Leadership

*R* = 0.695; *R*^*2*^ = 0.483

*b*^a^ Unstandardized beta coefficient

^*^p < 0.001

^**^p < 0.05

As seen in Table 2, all variables included in the regression analysis explain 48.3% (*R*^*2*^ = 0.483) of the change in psychological resilience. It was determined that empowering leadership behavior had a positive and significant effect on psychological resilience (b = 0.479, 95% Confidence Interval [0.218, 0.740], *p* < 0.001); while gender had a negative but insignificant effect (b = -0.046, 95% Confidence Interval [-0.185, 0.094], *p* > 0.05). The interactive effect (moderating effect) of empowering leadership and gender variables on psychological resilience was found to be significant (b = 0.196, 95% Confidence Interval [0.028, 0.365], *p* < 0.05). This finding supports the H2 hypothesis of the study. It was observed that the level of education (b = 0.009, 95% Confidence Interval [-0.077, 0.095], *p* > 0.05), educational status (b = -0.148, 95% Confidence Interval [-0.375, 0.080], *p* > 0.05), and marital status (b = -0.058, 95% Confidence Interval [-0.213, 0.097], *p* > 0.05), which were analyzed as control variables, did not have significant effects on psychological resilience in the established model.

**Fig. 1:**
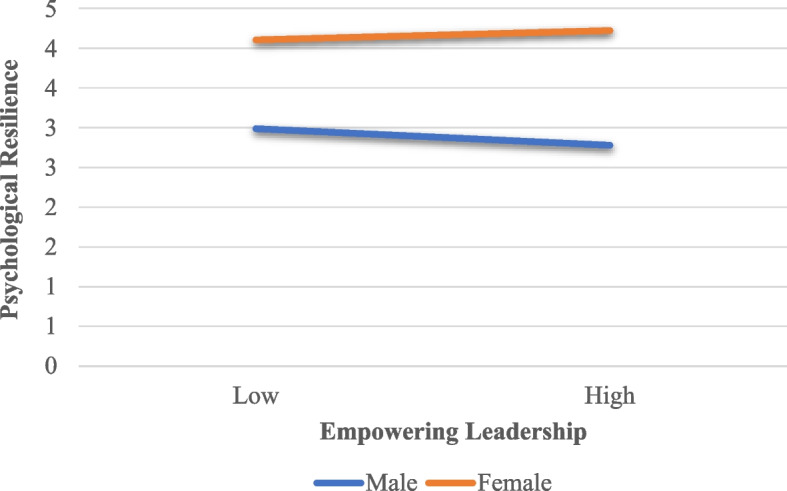
Simple slope graph by gender

In order to better understand the moderating effect of gender on the relationship between empowering leadership and psychological resilience, a simple slope graph was drawn (see Table 3 and Fig. 1).

**Table 3:** The effects of empowering leadership on psychological resilience by gender

Variables	*b*	S.E	95% Confidence Interval Minimum	95% Confidence Interval Maximum
Male (1)	0.675	0.059	0.560	0.790
Female (2)	0.872	0.063	0.749	0.995

*b* Unstandardized beta coefficient, *S.E*. Standard error

When the moderating effect of gender is examined based on Table 3 and Fig. 1, it is understood that the effects of empowering leadership on psychological resilience differ in male and female teachers. In other words, empowering leadership significantly affects psychological resilience in both male (b = 0.675, 95% Confidence Interval [0.560 0.790], *p* < 0.001) and female (b = 0.872, 95% Confidence Interval [0.749, 0.995], *p* < 0.001) teachers. However, the positive effect of empowering leadership behaviors of school principals on the psychological resilience of female teachers is stronger compared to male teachers.

The findings of the regression analysis conducted to test the moderating effect of the length of time spent with the school principal on the effects of the empowering leadership behaviors of school principals on the psychological resilience of teachers are presented in Table 4.

**Table 4:** Regression analysis findings regarding the moderating effect of length time spent with the school principal (*n* = 362)

Variables	*b*^*a*^	S.E	t	p	95% Confidence Interval	
					Minimum	Maximum
Constant	3.745	0.207	18.123	0.000^*^	3.338	4.151
EL	0.756	0.043	17.629	0.000^*^	0.672	0.841
Time	-0.003	0.023	-0.108	0.914	-0.049	0.043
EL x Time	0.077	0.027	2.871	0.004^**^	0.024	0.129
Level of Education	0.004	0.044	0.082	0.935	-0.082	0.089
Educational Status	-0.160	0.115	-1.386	0.167	-0.386	0.067
Marital status	-0.019	0.077	-0.248	0.804	-0.172	0.133

*S.E.* Standard Error, *EL* Empowering Leadership, *Time* Length of time spent with the school principal

*R* = 0.698; *R*^*2*^ = 0.487

*b*^a^ Unstandardized beta coefficient

^*^p < 0.001

^**^p < 0.01

As seen in Table 4, all variables included in the regression analysis explain 48.7% (*R*^*2*^ = 0.487) of the change in psychological resilience. It was found that empowering leadership behavior had a positive and significant effect on psychological resilience (b = 0.756, 95% Confidence Interval [0.672, 0.841], *p* < 0.001); while the length of time spent with the school principal had a negative but insignificant effect (b = -0.003, 95% Confidence Interval [-0.049, 0.043], *p* > 0.05). The interactive effect (moderating effect) of empowering leadership and length of time spent with the school principal on psychological resilience was determined to be significant (b = 0.077, 95% Confidence Interval [0.024, 0.129], *p* < 0.01). This finding supports the H3 hypothesis of the study. It was determined that the level of education (b = 0.004, 95% Confidence Interval [-0.082, 0.089], *p* > 0.05), educational status (b = -0.160, 95% Confidence Interval [-0.386, 0.067], *p* > 0.05), and marital status (b = -0.019, 95% Confidence Interval [-0.172, 0.133], *p* > 0.05), which were analyzed as control variables, did not have significant effects on psychological resilience in the established model.

**Fig. 2:**
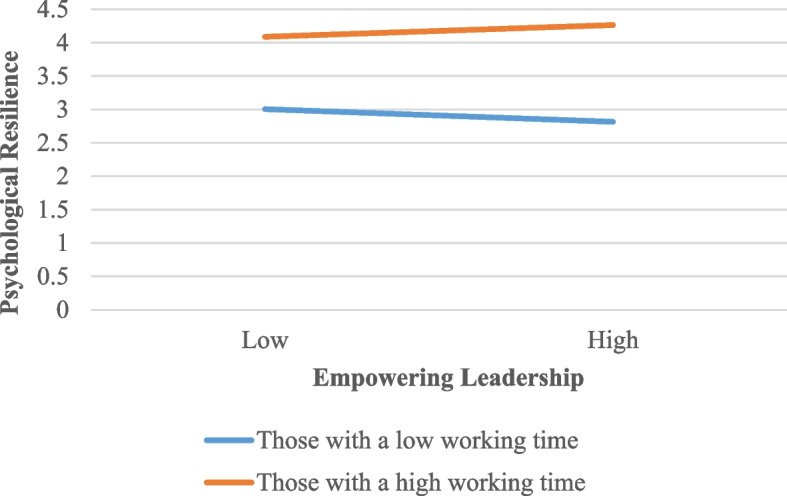
Simple slope graph by the length of time spent with the school principal

In order to better understand the moderating effect of the length of time spent with the school principal on the relationship between empowering leadership and psychological resilience, a simple slope graph was drawn (see Table 5 and Fig. 2).

**Table 5:** The effects of empowering leadership on psychological resilience by the length of time spent with the school principal

	*b*	S.E	95% Confidence Interval Minimum	95% Confidence Interval Maximum
-1 Standard Deviation	0.652	0.058	0.538	0.766
Mean	0.756	0.043	0.672	0.841
+ 1 Standard Deviation	0.873	0.057	0.761	0.985

*b* Unstandardized beta coefficient, *S.E.* Standard error

When the moderating effect of the length of time spent with the school principal is examined according to Table 5 and Fig. 2, it is seen that the effects of empowering leadership on psychological resilience differ in teachers who worked with the school principal for a short period of time and those who worked with the school principal for a longer period of time. In other words, empowering leadership significantly affects psychological resilience in teachers whose length of time spent with the school principal is both short (b = 0.652, 95% Confidence Interval [0.538, 0.766], *p* < 0.001) and longer (b = 0.873, 95% Confidence Interval [0.761, 0.985], *p* < 0.001). Additionally, it was found that the positive effect of empowering leadership behaviors of school principals on the psychological resilience was stronger in teachers who had a longer length of time spent with the school principal compared to teachers who had a short length of time spent with the school principal.

## Discussion

This study aimed to investigate the relationship between empowering leadership and psychological resilience of teachers in schools by testing a model that takes into account gender of teachers and length of time they spent with the school principals in the context of Siirt province of Türkiye. Our findings show that school principals had high empowering leadership behaviors and psychological resilience according to teachers' perceptions in the context of Siirt province. This finding is promising for Siirt province, where teacher circulation is high. It is likely that school principals are especially aware of the difficulties that newly appointed teachers experience/may experience. It can be said that principals take care to empower and support teachers so that the education-teaching process at school is not negatively affected by these problems. In terms of the centralized Turkish education system, the fact that school principals adopted empowering leadership behaviors can be interpreted as the reform initiatives are effective and empowering leadership behaviors will find a response in Turkish schools. Although it is known in the literature that teacher psychological resilience contributes significantly to school outcomes [131, 132], the number of studies on the effects of principal leadership on teacher psychological resilience is low [39, 133]. Therefore, considering this need, we examined the effects of empowering leadership on teacher psychological resilience. We discovered a significant relationship between empowering leadership and psychological resilience in the Turkish school context. [134], who discovered the significant relationship between empowering leadership and teacher agency in their study conducted in the Turkish sample, attributed this result to the fact that Turkish culture has not yet moved beyond traditional and feudal habits, and therefore principals still assign teachers the role of nurturing 'mother' or 'father'. Therefore, our findings contributed to the EDML literature and produced results compatible with traditional Turkish culture, where the empowering school principal style strengthens teachers [37] and increases their resilience [39].

Our study results confirmed H2 by showing that the gender variable has a moderating effect between empowering leadership behaviors of school principals and psychological resilience of teachers. Spreitzer [135] explained this situation by saying that empowerment is context-specific. In other words, empowering behaviors of school principals may not create the same sense of empowerment in every teacher. For example, cultural factors are context-specific. Türkiye is an eastern country where the patriarchal approach is dominant [65]. In such societies, the role of men is emphasized as being assertive, durable, and focused on material success, while women are more modest, sensitive, and concerned with quality of life. Studies conducted in Türkiye show that psychological resilience perceptions of male teachers are higher than those of female teachers [136, 137]. In Türkiye, while boys may be more resilient because they work in heavier jobs from a young age and have to cope with difficulties, girls work more at home [138]. Therefore, when they have a career, men can be more resilient, while women may need more support. For this reason, in countries where masculine culture is dominant, as in the case of Türkiye, gender roles and attitudes can affect psychological resilience based on leadership style differently than in other countries. The findings contribute to the literature of studies that show gender as a moderator in the psychological effects of leaders on followers [139, 140].

Our study confirmed H3 by proving the moderating effect of the length of time spent with the school principal on the positive effect of school principals on psychological resilience of teachers. Empowering leadership positively predicts psychological resilience, which may lead to positive behaviors and outcomes, which may increase willingness of teachers to work longer at the school. Therefore, the length of time spent with the school principal is supportive of empowering leadership and psychological resilience. Özer and Çelikten [113] finding that the teachers who had a longer total working time with the principal in the school they worked at perceived empowering leadership at a higher level compared to the teachers who had a shorter total working time is parallel to that of our study. Moreover, there are also findings in the literature that as the total working time with the principal increases, the principal becomes more closely known by teachers, teacher-principal relationships become stronger, trust in the principal increases, and therefore empowering leadership perceptions of teachers increase [92, 141]. In fact, [47] found that increased length of time spent with the school principal reduced the negative effects of abusive leadership on teacher motivation. However, there is also evidence in the literature from studies conducted in different countries that negative outcomes occurred as the length of time spent with the school principal increased. For example, [46] study in the context of Spanish secondary schools discovered that working with the school principal for long periods of time harmed motivation of teachers. Westphal and Fredrickson [142] explained these results with the behavior change of the principals over time, [143] explained them with the principal's adherence to old models and not being open to innovations. In fact, the relationship of our study with other findings in the literature points to the importance of contextual factors, school culture, and leadership styles. In other words, the positive effect of the length of time spent with the school principal in our study may be due to the school culture and the dynamics of teacher-principal interaction.

On the other hand, the education systems, cultural norms, and school structures of different countries may also lead to different effects of the same variables on the relationship between the empowering leadership of school principals and the psychological resilience of teachers. As a matter of fact, previous studies revealed that the effect of empowering leadership on various positive teacher attitudes is realized through various mediating variables such as teacher self-efficacy, trust in the principal, and psychological empowerment [92, 144–146] This study made an original contribution to the literature by proving the moderating role of demographic variables such as gender and length of time spent with the school principal.

## Theoretical implications

This study has made important theoretical contributions to the literature. First, consistent with previous literature, our study revealed statistically significant relationships between empowering leadership of principals and resilience capacity of teachers. This result extends the literature confirming the approach that supportive behaviors of leadership will make employees more resilient to challenges in the context of social exchange theory [146]. Second, previous researchers typically focused on the impact of leadership approaches on various teacher outcomes [147]. Our study provides a new perspective on how leadership empowering behavior can affect teacher resilience through moderation by gender and the length of time spent with the school principal and provides a better understanding of the relationship. This study showed that female teachers were more affected by the empowering leadership behaviors of school principals than male teachers and that this influence increased their psychological resilience. This finding provides an important contribution to the theoretical literature on the moderating role of gender. Third, one of the important theoretical implications of the fact that empowering leadership behaviors perceived by teachers supported their psychological resilience as the length of time teachers worked with the school principal increased is that it emphasizes that the results of the relationship between leader-member exchange, which may depend on various contextual factors, may differ. More importantly, we discovered the importance of contextual factors, including principals' behaviors, in terms of demographic variables.

## Practical implications

Our findings present practical implications for educational leaders and policy makers. First, educational leaders who recognize the positive effects of their empowering behaviors on teacher psychological resilience should strive to adopt leadership practices prioritizing supportive, empowering, and accountable interactions. However, school principals do not have sufficient opportunities in terms of legislation and resources. Therefore, it can be said that school principals need various authorities and resources to create structures that will empower teachers. Recent reform movements in Türkiye also emphasize that school principals should support teachers [51, 148]. For this reason, we recommend that principals be given more autonomy and authority in countries with centralized education systems such as Türkiye. In this context, legal regulations to be made regarding the authority and responsibilities of school principals can support empowering leadership practices in schools. Secondly, leadership development programs can encourage positive leadership behaviors by focusing on encouraging the empowerment of school principals and emphasize the importance of supportive interactions with teachers. Particularly in more disadvantaged schools, where new teachers form the majority, the school principals’ empowering behaviors may benefit the resilience of teachers and therefore they may need more training in empowering leadership behaviors. Current studies show that principals only value the effectiveness of instructional, transformational, and visionary leadership behaviors [149], which highlights the lack of programs that are much needed and specifically target empowering leadership behaviors that positively impact interpersonal interactions. School principals can be encouraged and supported to participate in in-service activities aimed at developing their empowering leadership competencies and increasing their awareness. To this end, policy makers can emphasize the implementation of training programs and seminars that will enable principals to exhibit empowering behaviors. For example, [150] found that school administrators receiving “mindfulness” training increased resilience of teachers. Similarly, it is recommended that such programs be context-sensitive and pay more attention to how historical, social, political, and structural environmental conditions support leadership models [151]. Additionally, considering the moderating effect of gender, school principals should take care to establish empowering bonds with male teachers as well as with female teachers. Additionally, school leaders should be aware of the importance of empowering bonds for female teachers as a mechanism of influence in increasing psychological resilience.

## Limitations of the study and suggestions for future research

It is useful to take some limitations into consideration when interpreting and evaluating the results of the study. One of the most important limitations of the study is that the sample was collected from only one province. For this reason, the results of a study conducted with a wider range of participants from different provinces may differ from the results of the current study. Empowering leadership and level of education variables, which are among the variables of the study, are accepted as school level variables. However, single-level analyses were used in the study. The study data were collected through online forms. Therefore, information regarding which schools the variables were related to could not be obtained. This situation led to single-level analyses instead of multi-level analyses. Although it is reliable that the empowering leadership behaviors of school principals were measured based on the perceptions of teachers in the study, measuring leadership behaviors according to the perceptions of both teachers and school principals may produce different results. Since the study was designed as cross-sectional, the relationships between the variables cannot be considered as a full cause-effect relationship. The causal relationship between empowering leadership and other variables can be revealed through longitudinal and experimental studies. Both scales used in the study are unipolar Likert type scales. Bipolar Likert scales can be used to make more accurate and reliable predictions in future studies. On the other hand, using only scales in the study can be considered as another limitation. The results of the study can be examined in more depth and detail using different data collection techniques such as interview and observation in accordance with qualitative or mixed method research methods. Finally, individual variables such as age and tenure were not controlled in this study. The possible effects of these variables on the study results should be taken into consideration. It is recommended that future studies increase the generalizability of the results by including such individual variables in the model.

## Conclusion

This study, based on social exchange theory, confirmed that there is a significant relationship between EL and teacher PR, as predicted. The results also showed moderating effects of gender and length of time spent with the school principal on the relationship between empowering leadership and psychological resilience of teachers. Compared with previous studies, these moderating effects discovered in our study made an original contribution to the literature. These results are important for understanding the relationships between school-level (i.e., leadership) and teacher-level variables (i.e., gender and length of time spent with the school principal) that play a role in teacher resilience. This effect, which emerged in the context of Siirt, provides a unique contribution in terms of education services in all provinces where new teacher appointments and teacher circulation are intense, especially in the Eastern and Southeastern Anatolia Regions of Türkiye, and in countries with similar culture and social structure.

## Supplementary Information

Supplementary Material 1.

## Data Availability

The raw data supporting the conclusions of this article will be made available by the authors, without undue reservation, to any qualified researcher.
